# Erratum zu: „Terror awareness“ bei Humanmedizinstudierenden im 5. Jahr des Mannheimer Reformierten Curriculums Medizin plus

**DOI:** 10.1007/s00113-021-01027-1

**Published:** 2021-07-07

**Authors:** Arnold J. Suda, Isabel Höppchen

**Affiliations:** 1 AUVA Unfallkrankenhaus Salzburg, Akademisches Lehrkrankenhaus der Paracelsus Universität, Doktor-Franz-Rehrl-Platz 5, 5010 Salzburg, Österreich; 2 Medizinische Fakultät Mannheim der Universität Heidelberg, Orthopädisch-Unfallchirurgisches Zentrum, Universitätsmedizin Mannheim, Mannheim, Deutschland; 3 Abteilung Allgemeinmedizin und Versorgungsforschung, Universitätsklinikum Heidelberg, Heidelberg, Deutschland

**Erratum zu:**

**Unfallchirurg 2020**

10.1007/s00113-020-00808-4

Der Artikel „‚Terror awareness‘ bei Humanmedizinstudierenden im 5. Jahr des Mannheimer Reformierten Curriculums Medizin plus“ von Arnold J. Suda und Isabel Höppchen wurde ursprünglich am 28.04.2020 ohne „Open Access“ online auf der Internetplattform des Verlags publiziert. Die Autoren haben sich jedoch nachträglich für eine „Open Access“-Veröffentlichung entschieden. Das Urheberrecht des Artikels wurde deshalb am 06.05.2021 in © Der/Die Autor(en) geändert.

